# Perception of traumatic childbirth of women, influencing factors and its relationship with post-traumatic stress disorder

**DOI:** 10.3389/fpubh.2024.1485766

**Published:** 2025-01-17

**Authors:** Weiwei Wei, Xiaoyan Feng, Hong Qin, Xiaochang Yang

**Affiliations:** ^1^Obstetrical Department, The First Affiliated Hospital of Chongqing Medical University, Chongqing, China; ^2^Nursing Department, The First Affiliated Hospital of Chongqing Medical University, Chongqing, China

**Keywords:** psychological birth trauma, postpartum post-traumatic stress disorder, relationship, factors, investigation, research

## Abstract

**Objectives:**

Some of individuals with psychological birth trauma (PBT) develop into postpartum post-traumatic stress disorder (PP-PTSD) further. Study investigated the prevalence and influencing factors of PBT and its correlation with PP-PTSD, to fill the literature gap in the prevention of postpartum related psychological birth trauma.

**Methods:**

A total of 306 women who gave birth vaginally from Chongqing, China participated in this study. Pregnant women filled the basic information when they entered the delivery room and waited for delivery. The psychological birth trauma and posttraumatic stress scales were completed during 1–3 days postpartum. Information on labor and delivery outcomes is available in the hospital's electronic medical record. Variation analysis and Pearson correlation were used on the influencing factors of PBT and the correlation with PP-PTSD.

**Results:**

The median PBT score of the subjects in this study was 41 points; the incidence rate of PTSD (scores greater than 38) is 5%, with an average score of (22.38 ± 7.126). All dimensions of PBT positively correlated with post-traumatic stress disorder, respectively. Lower score of perceived PBT is associated with work, exercise and learning about delivery; is also associated with less vaginal examinations, the use of pain relief and doula accompaniment.

**Conclusion:**

This study suggests that every dimension of PBT should be taken seriously to prevent PP-PTSD. Work, exercise and learning about delivery during pregnancy may be promising protective factors for perceived PBT; the use of pain relief or doula accompaniment are still effective ways to Improve the delivery experience.

## 1 Introduction

Postpartum post-traumatic stress disorder (PP-PTSD) refers to delayed psychological stress disorder that occurs in postpartum women after childbirth (1). The positive detection rate of PP-PTSD in China was 6.57%to 11.6% (2–4), higher than the incidence according to meta analysis among community samples increasing from 3.17% (5) to 4% (6). The symptoms includes traumatic memory, cognitive negativity, and avoidance behavior (7), associated with a lower rate of initiation of breastfeeding (8) and poor child social-emotional development (9) in the long term. Mothers who have no fixed job, vaginal delivery, or experience discomfort during pregnancy are more likely to suffer from PP-PTSD (10).

As a phenomenon associated with the occurrence of PP-PTSD, the research on psychological birth trauma (PBT) began in 1995, and the peak period in this field is predicted to be in 2026 (11). PBT refers to the negative feelings that due to events directly or indirectly related to childbirth that emphasizes the subjective feeling of postpartum women (12). Severe labor pain, premature birth, emergency cesarean section, difficult delivery, and the use of suction devices or forceps for delivery are known influencing factors of it (13). PBT can lead to women feeling out of control, depressed, anxious (14), a minority of them will develop PP-PTSD (15, 16). Professor Beck (17) used the word “ripple effect” to describe the negative impacts of PBT, but there is the potential to prevent postpartum PTSD by maternity care to keep women from PBT (18).

Research on PBT in China has been slow to develop since 2017, and the recent review failed to discuss the incidence of PBL or intervention methods in China (11). There is no original research in China on mothers' perception of PBT or its relationship with PP-PTSD. Therefore, the present study investigated the prevalence and influencing factors of PBT in Chongqing, China, as well as the correlation between PP-PTSD and perceived PBT to fill the literature gap of this field.

## 2 Methodology

### 2.1 Participants

This study used convenience sampling method and selected postpartum women who underwent vaginal delivery at a hospital in Chongqing from February to April 2024 as the research subjects. Women who experienced cesarean section or neonatal death were excluded from the study. Questionnaire Star was employed on questionnaire collection twice. Pregnant women filled the basic information when they entered the delivery room and waited for delivery. Then completed the scales during 1–3 days postpartum. Information on labor and delivery outcomes is available in the hospital's electronic medical record. All participants signed an informed consent. After repeated attempts by the researchers, it takes at least about 100 seconds to fill in the questionnaire, so this study excluded questionnaires with <100 seconds to prevent subjects from filling in the questionnaire without reading the questions. A total of 306 valid questionnaires were included in the analysis.

### 2.2 Measures

#### 2.2.1 Psychological birth trauma

The Scale for Measuring Birth Trauma Perception with Women Who Deliver Vaginally was administrated to all participants during 1–3 days postpartum. This scale was developed by Lian (19) and is suitable for the cultural, reproductive, and medical backgrounds of China, specifically for women who have delivered vaginally. The scale includes four dimensions: medical support trauma perception, delivery pain trauma perception, family support trauma perception, and delivery outcome trauma perception, with a total of 31 items. The scale has good reliability and validity and can be used as a measurement tool for women undergoing natural childbirth (20). The scale was only used once in Jiangxi Province, China by the founder of the scale, and the median score of the subject group was 50, but the PBT positive rate or positive score line was not discussed (19).

#### 2.2.2 Postpartum post-traumatic stress disorder

PP-PTSD was assessed using The Civilian Version of the Posttraumatic Stress Inventory (PCL-C) during 1 to 4 months postpartum, which was developed by Ruggiero et al. (21) based on the 4th edition of the Diagnostic and Statistical Manual of Mental Disorders. This is a self-report questionnaire consisting of 17 items with a score range of 17–85 points. The higher the score, the higher the risk or severity of PSTD in the individual. Total score exceeds 38 points indicates the existence of PP-PTSD. Yang et al. (22) sinicized and applied it in 2007; Wang et al. (2) supported the use of PCL-C in China; Wang et al. (3) used the scale to measure the occurrence of postpartum PTSD.

## 3 Control variables and data analysis

Control variables included two parts: Demographics data (age, education level, work situation, and marital relationship), pregnancy and childbirth data (pregnancy exercise, parity, planned pregnancy, infant gender expectations, planned delivery methods, participation in prenatal courses, level of understanding of delivery methods, husband's understanding of delivery knowledge, planned feeding methods, whether painless delivery or doula accompaniment is used, and the willing to wait and deliver together with other mothers).

SPSS 23.0 statistical software is employed for data analysis. Normally distributed econometric data are described as mean ± standard deviation, and inter group comparisons are conducted using independent sample *t*-tests and analysis of variance. Count data is described in terms of examples and percentages. Pearson correlation analysis explores the correlation between perceived childbirth trauma and post-traumatic stress disorder. Perform multiple linear regression analysis on items with *P* < 0.05 in the uni-variate analysis, with a testing level of α = 0.05.

## 4 Results

The characteristics and demographics of the participants are shown in Table 1. The data analysis include data from 306 mothers age ranged from 22 to 41 years old (30.14 ± 3.51 y) who gave birth naturally. The median PBT score of the subjects in this study was 41 points, which was lower than the survey data in Jiangxi (19) (median 50 points); the incidence rate of PP-PTSD (scores >38) is 5%.

**Table 1:** Characteristics of the study population.

**Item**	**Group**	***N* (%)**
Age	< 30 years old	133 (43.5)
	≥30 years old	173 (56.5)
Education	High school or below	37 (12.1)
	Junior college	97 (31.7)
	College	146 (47.7)
	Master degree or above	26 (8.5)
Work situation	Individual merchants	9 (2.9)
	Freelance	55 (18.0)
	Unemployed	17 (5.6)
	Public institutions or officials	225 (73.5)

As for psychological birth trauma, the median score is 41 while average score is (43.37 ± 9.46), the scores for each dimension are shown in Table 2. In terms of demographic characteristics, there was no significant difference in PBT scores between women aged 30 years or older and those aged < 30 years, but there were significant differences between primipara and parturient women. The score range for PP-PTSD is 17–56, with an average score of (22.38 ± 7.126). The Pearson correlation analysis results showed that the scores of the four dimensions of perceived childbirth trauma were positively correlated with the total score of post-traumatic stress disorder, as shown in Table 3.

**Table 2:** Score of BPT.

**Dimension**	**Number of item**	** $\bar{X}\pm S$ **
Trauma on medical support	14	15.77 ± 3.76
Trauma on labor pain	7	12.98 ± 4.39
Trauma on family support	6	6.45 ± 1.38
Trauma on delivery outcome	4	8.17 ± 3.48

**Table 3:** Correlation analysis between four dimensions and PTSD (r value).

**Item**	**Trauma on medical support**	**Trauma on labor pain**	**Trauma on family support**	**Trauma on delivery outcome**
PTSD	0.307^**^	0.383^**^	0.311^**^	0.357^**^

^**^P < 0.01.

The results of single factor analysis show that protective factors during pregnancy were knowledge about delivery, husband's initiative in learning childbirth knowledge, participation in pregnancy school, work and exercise during pregnancy, plan of breastfeeding, and the lack of complications. The risk factors during birth were wish to wait and give birth without other mothers, without pain relief or doula accompaniment. See in Table 4.

**Table 4:** Univariate analysis of perception of childbirth trauma (*n* = 306).

**Item**	**Types**	***N* (%)**	**Score of BPT (mean ±SD)**	**Statistical value**	**P**
Delivery experience	Primipara	218 (71.2)	44.46 ± 9.83	t = 3.55	< 0.001
	Multiparous	88 (28.8)	40.66 ± 7.88		
Duration of the first stage of delivery	< 10 h	183 (59.8)	42.38 ± 8.67	t = −2.175	0.031
	≥10 h	123 (40.2)	44.85 ± 10.39		
vaginal examination frequency	1-3	162 (52.9)	41.96 ± 7.97	F = 6.933	0.001
	4-6	92 (30.1)	43.53 ± 8.10		
	≥7	52 (17.0)	47.46 ± 13.26		
Medical intervention	Yes	86 (28.1)	46.29 ± 11.06	t = 3.44	0.001
	No	220 (71.9)	42.23 ± 8.51		
Work during pregnancy	Yes	188 (61.4)	42.21 ± 8.67	t = −2.59	0.010
	No	118 (38.6)	45.21 ± 10.63		
Exercise during pregnancy	Yes	153 (50.0)	41.34 ± 8.23	t = −3.84	< 0.001
	No	153 (50.0)	45.40 ± 10.17		
Expectant feeding method	Breast feeding	191 (62.4)	42.35 ± 8.76	F = 3.210	0.042
	Formula feeding	9 (2.9)	43.22 ± 6.61		
	Mixed feeding	106 (34.6)	45.23 ± 10.72		
Understanding about childbirth	Almost unknown	24 (7.98)	45.67 ± 11.03	F = 9.150	< 0.001
	Partial	196 (64.1)	44.66 ± 9.80		
	Familiar	86 (28.1)	39.79 ± 7.02		
Receiving delivery education	Yes	63 (20.6)	39.03 ± 5.47	t = −5.82	< 0.001
	No	243 (79.4)	44.49 ± 9.94		
Partner's activity on childbirth knowledge	Active	188 (61.4)	40.03 ± 7.48	t = −8.189	< 0.001
	Inactive	118 (38.6)	48.69 ± 9.85		
pregnancy complications	Yes	199 (65)	44.16 ± 10.07	t = 2.007	0.046
	No	107 (35)	41.90 ± 8.03		
Use of labor analgesia	Yes	171 (55.9)	41.88 ± 8.16	t = −2.937	0.004
	No	135 (44.1)	45.08 ± 10.54		
Use of Doula accompaniment	Yes	155 (50.7)	40.15 ± 6.84	t = −6.373	< 0.001
	No	151 (49.3)	46.67 ± 10.59		

A multiple linear regression analysis was conducted on the influencing factors of perceived PBT in women, with the total score of postpartum trauma perception as the dependent variable and statistically significant indicators in uni-variate analysis as independent variables. The specific assigned values are shown in Table 5. The factors that were ultimately included in the regression equation include pregnancy exercise, husband's initiative on childbirth knowledge, number of vaginal examinations, expected feeding methods, the use of doula accompaniment and the understanding of childbirth methods. The adjustment determination coefficient R2 of the fitting model is 0.442, indicating that the fitting model can analyze 44.2% of the variability. The overall test of the fitting model is *P* < 0.05, indicating that the fitting effect of the model is good, as shown in Table 6. Compared to pure breastfeeding, mothers who plan to mix feed have more severe childbirth trauma; the more you understand your delivery method, the lighter the trauma of childbirth; Mothers who have undergone 11 or more vaginal examinations perceive more severe childbirth trauma than mothers who have undergone < 5 vaginal examinations.

**Table 5:** Variable assignment.

**Variable**	**Assigned method**
Perceived PBT Score	Original numerical values
Work during pregnancy	Yes=1, No=2
Exercise during pregnancy	Yes=1, No=2
Partner's activity on childbirth knowledge	Yes=1, No=2
Use of labor analgesia	Yes=1, No=2
Use of doula accompaniment	Yes=1, No=2
Medical intervention	Yes=1, No=2
Delivery frequency	Primipara = 1, Multiparous = 2
Duration of the first stage of delivery	< 10 h = 1, ≥10 h = 2
Vaginal examination frequency	1–5 = Dummy variable, 6–10 = (1, 0), 11 and above = (0, 1)
Expectant feeding method	Breast feeding = Dummy variable, Formula feeding = (1, 0), Mixed feeding = (0, 1)
Understanding about childbirth	Almost unknown = Dummy variable, Partial = (1, 0), Familiar = (0, 1)

**Table 6:** Multiple linear regression analysis of perception of childbirth trauma (*n* = 306).

**Independent variable**	**Regression coefficient**	**Standard error**	**Standardized coefficient**	**t**	***P***
(Constant)	10.414	5.277		1.974	0.049
Exercise during pregnancy	3.089	1.006	0.164	3.070	0.002
Partner's activity on childbirth knowledge	4.087	0.984	0.211	4.154	< 0.001
Use of doula accompaniment	4.640	0.938	0.246	4.945	< 0.001
**Vaginal examination frequency (refer to 1–5)**					
6–10	1.192	1.165	0.058	1.024	0.307
11 and above	4.382	1.471	0.174	2.978	0.003
**Expectant feeding method (refer to Breast feeding)**					
Formula feeding	2.645	2.535	0.047	1.043	0.298
Mixed feeding	2.775	0.952	0.140	2.915	0.004
**Understanding about childbirth (refer to Almost unknown)**					
Partial	−1.152	1.665	−0.059	−0.692	0.490
Familiar	−4.079	1.859	−0.194	−2.194	0.029

## 5 Discussion

### 5.1 Main findings

The purpose of this study is to make up for the research gap in the field of PBT in China among vaginal maternity mothers, and to discuss the influencing factors, protective factors and the relationship between PBT and PTSD. The results are reassuring that PBT level was associated with PP-PTSD and breastfeeding intention. Exercise, work and childbirth knowledge during pregnancy were protective factors for PBT. The use of labor analgesia and doula accompaniment can improve the labor experience from a medical support level.

The survey conducted by the scale founder (19) among 193 people in Jiangxi Province on childbirth trauma showed that the median score is 50. Data from 306 participants in this study shows a median score of 41 and the average is (43.37 ± 9.46). The average score of PP-PTSD is (22.38 ±7.126). As cesarean is an independent risk factor for PP-PTSD (23), the subjects in this study were all vaginal mothers, which made their scores relatively low. In terms of demographic characteristics, PBT scores were not significantly affected by age, but there were significant differences in PBT scores between primipara and parturient women. The range of the psychological pain experience rate during childbirth is (9%−44%) (24). A cross-sectional survey in the Netherlands found that 9.1% of 907 women have experienced childbirth trauma (15). A study of 400 women in Iran reported a rate of 54.5% experienced childbirth trauma; lower socio-economic status (25). There is a lack of unified screening standards for PBT in the world, different measurement tools lead to different understandings of the incidence of PBT.

There are four dimensions in PBT perception scale including medical support, childbirth pain, family support, and perception of childbirth outcome trauma. Trauma from medical support and pain is more serious than family support or delivery outcome, consistent with the Jiangxi Province study (19). All dimensions are positively correlated with PP-PTSD respectively. The incidence rate of PP-PTSD from present data is 5.0%, which is higher than the meta-analysis data in 2014 (3.17%) (5) and 2017 (4.0%) (6). Although most studies evaluating both PP-PTSD and childbirth trauma have only reported the independent incidence (15, 16), recent reviews suggest that assessing childbirth as a possible trauma is an important risk factor for PP-PTSD (26). The results of this study suggest that every dimension of childbirth trauma experience deserves attention to prevent the development of PP-PTSD. Besides, mothers who expect to breastfeed have lower scores of BPT compared to those who expect to use mixed feeding methods. This finding is consistent with the result that high traumatic childbirth perception level relates to low breastfeeding self-efficacy (27). Expectations about feeding methods, especially breastfeeding self-efficacy, is influenced by a mother's belief in her ability to breastfeed her infant (28), and further related to BPT level.

The current analysis shows that participating in work and exercise during pregnancy may help improve the delivery experience. Regular physical exercise during pregnancy decreases incidence of gestational diabetes, hypertensive disorders (29), reduces the odds of prenatal depression (30). The reason may be the effect of exercise to facilitate emotional recovery (31). Previous finding shows that a small decrease in birth weight was seen among women who left work prior to term but not among those who worked all 9 months (32). It is good for mothers to try various sports that are suitable for pregnancy during pregnancy. Previous studies have focused on the changes of Chinese people's work behavior during pregnancy (33), but the impact of working persistence during pregnancy has been ignored. In addition, receiving prenatal education and understanding childbirth knowledge are also beneficial, previous studies have also advocated strengthening prenatal learning (34), which improves delivery self-efficacy (35). Remarkably, the partner's active learning of childbirth knowledge significantly affects the childbirth experience of mothers. The literature has also pointed out the importance of improving the mastery of pregnancy and childbirth knowledge for partners, which means they empathize with and value their wives (25). Meta-analyses also revealed that interventions including delivery education can reduce the fear of childbirth (36, 37).

Other factors includes complications, labor analgesia, and vaginal examination frequency seem to influence the score of PBT. Prior literature has also proposed that pregnancy complications Pregnancy complications are associated with short- and long-term psychologic sequelae, including depression, anxiety, and PTSD (38). Labor pain also plays a role in the development of PP-PTSD (39), while Pharmacological and non-pharmacological pain relief methods may help women feel in control (40). Present results suggest to control the number of vaginal examination before delivery to 1 to 5 times. The ideal vaginal examination frequency is once every 4 h for low-risk women as The World Health Organization (WHO) recommended (41); it was also found that vaginal examination performed by the same person resulted in more positive experiences for mothers (42). If women can be given more sense of control and empowerment at the level of medical intervention, their self-efficacy and self-esteem would increase after a pleasant childbirth (43). The hospital where this study was conducted has set up midwife clinics and breastfeeding clinics to help mothers prepare for childbirth and improve breastfeeding rates. The maternity school allows pregnant women and their families to accept prenatal education for improving the delivery experience.

### 5.2 Strengths and limitations

This study aims to fill the gap of PBT research in China, innovatively considered the impact of work, exercise and learning childbirth knowledge during pregnancy on the delivery experience. An significance of the current study is to provide evidence for the relationship between perceived PBT level and the expectation of breastfeeding.

The results of this study were limited to the vaginal parturient population in Chongqing, China. One of the limitations of this study is that it did not classify and discuss the types and intensity of exercise during pregnancy. To draw clinically relevant conclusion, empirical studies need to consider specific types and intensity of exercises in larger samples. Another limitation of the present study is that the proportion of patients who further develop PP-PTSD was not monitored in a population with high PBT score. It is important to track the individuals who have experienced severe childbirth trauma. It can be further explored whether individuals' perception of PBT and their attitudes toward PBT increase or decrease the risk of PP-PTSD. This study did not collect a baseline of prenatal depression and PTSD before delivery, which should be considered in future studies.

## 6 Conclusion

This study focuses on the perception of childbirth trauma among Chinese women and its influencing factors, as well as the correlation between perception of childbirth trauma and PP-PTSD, filled the literature gap. The four dimensions of perceived PBT are positively correlated with post-traumatic stress disorder, respectively. The results of this study support the impact of childbirth analgesia and doula accompaniment on reducing the childbirth trauma perception; emphasize the importance of reducing the frequency of vaginal examinations, receiving childbirth education for women during pregnancy. Meanwhile, this study found that work and exercise during pregnancy have a positive impact on improving the delivery experience as promising directions for future research.

## Data Availability

The original contributions presented in the study are included in the article/supplementary material, further inquiries can be directed to the corresponding author.
